# Dysbiosis of the nasal microbiome is associated with prospective acute exacerbation of COPD

**DOI:** 10.1186/s40168-026-02357-1

**Published:** 2026-03-16

**Authors:** Cristian Roca, Caleb C. Hemphill, Adam M. Speen, Ilona Jaspers, Matthew C. Wolfgang, M. Bradley Drummond

**Affiliations:** 1 Department of Microbiology and Immunology, University of North Carolina at Chapel Hill, Chapel Hill, NC USA; 2 Marsico Lung Institute/CF Center, University of North Carolina at Chapel Hill, 125 Mason Farm Road, 7105 Marsico Hall, Chapel Hill, NC 27599 USA; 3 Division of Pulmonary Diseases and Critical Care Medicine, University of North Carolina at Chapel Hill, Chapel Hill, NC USA; 4 Center for Environmental Medicine, Asthma, and Lung Biology, University of North Carolina at Chapel Hill, Chapel Hill, NC USA; 5 Department of Pediatrics, University of North Carolina at Chapel Hill, Chapel Hill, NC USA

**Keywords:** Pulmonary disease, Chronic obstructive; Immunity, Mucosal; Microbiome, Human

## Abstract

**Background:**

Acute exacerbation of chronic obstructive pulmonary disease (AECOPD) is often associated with respiratory viral infection and the need for increased medical intervention. The nasal mucosa plays a critical role in infection susceptibility and severity, with the nasal microbiome shaping mucosal immunity. This study investigated the association between the bacterial nasal microbiome and AECOPD. Participants included 41 individuals with COPD and 15 healthy non-COPD controls. Nasal microbiome composition was assessed from nasal epithelial lining fluid and compared at baseline between healthy participants and individuals with COPD, stratified by AECOPD history. COPD subjects who experienced one or more AECOPD in the year prior to enrollment were categorized as Ever AECOPD. COPD participants without a history of AECOPD in the prior year were categorized as Never AECOPD. Prospective exacerbation data were collected and used in a case–control analysis to identify clinical and microbiological markers predictive of future AECOPD in COPD-diagnosed subjects.

**Results:**

We found two distinct nasal microbiome architectures with enrichment of protective taxa (healthy signature) or pathobionts (pathogenic signature). Nasal microbiome analysis demonstrated significant differences in nasal bacterial composition between COPD-diagnosed individuals with prior AECOPD (Ever AECOPD) compared to healthy controls. For COPD individuals with no prior AECOPD (Never AECOPD), we identified two underlying community structures; Cluster 1 subjects harbored a nasal microbiome significantly similar to healthy controls (healthy signature) and Cluster 2 subjects were significantly similar to the Ever AECOPD cohort (pathogenic signature). We evaluated the baseline microbiome of COPD-diagnosed participants based on the occurrence of at least one AECOPD 1-year after baseline sample collection (prospective AECOPD) and found that the nasal microbiome was associated with the occurrence of future AECOPD events. Prospective exacerbation was associated with reduced relative abundance of *Dolosigranulum pigrum*. Further analysis by qPCR showed that decreased *D. pigrum* abundance was associated with lower lung function and higher risk of future AECOPD.

**Conclusions:**

Our data indicates that the nasal microbiome is associated with AECOPD phenotypes. Moreover, participants with decreased nasal *Dolosigranulum pigrum* abundance had lower lung function and a higher risk of future exacerbations. These findings suggest that *D. pigrum* may serve as a biomarker for AECOPD risk; however, validation of these findings in a larger multicenter cohort is needed.

Video Abstract

**Supplementary Information:**

The online version contains supplementary material available at 10.1186/s40168-026-02357-1.

## Introduction

Chronic obstructive pulmonary disease (COPD) is marked by episodes of intense worsening, known as acute exacerbations of COPD (AECOPD) [1]. Increased susceptibility to viral infection is considered a key factor contributing to AECOPD [2]; however, additional factors associated with AECOPD risk remain to be determined.

The nasal mucosa serves as the first line of defense against respiratory pathogens and plays a crucial role in determining susceptibility to and severity of respiratory infections [3]. The nasal microbiome plays a central role in regulating mucosal immunity and antimicrobial defenses [4, 5]. Growing evidence suggests that the nasal microbiome also influences the development and progression of lower airway diseases [6, 7]. In pediatric asthma, dysbiosis of the nasal microbiome has been linked to greater disease severity and more frequent exacerbations [8]. The presence of specific bacteria in the nasal cavity, such as *Moraxella catarrhalis* and *Haemophilus influenzae*, has been associated with asthma exacerbations [9, 10]. In addition, enrichment of pathogenic bacteria such as *Staphylococcus aureus* or a reduction of protective bacteria such as *Dolosigranulum pigrum* in the sinonasal compartment has been linked to respiratory diseases including pneumonia, asthma, and chronic rhinosinusitis [11–14]. While changes in the nasal microbiome have been associated with increased susceptibility to respiratory infections [15], an important trigger of AECOPD [16], a direct link between the nasal microbiome, COPD severity, and exacerbation risk has not been clearly established.

Although pulmonary inflammation and the lower airway microbiome have been studied in the context of AECOPD [17, 18], the relationship between the nasal microbiome and AECOPD remains poorly defined. Known risk factors for AECOPD are limited to prior exacerbations, older age, reduced lung function, and nasal colonization with methicillin-resistant *Staphylococcus aureus* (MRSA) [1, 19]. While MRSA colonization is a potentially treatable trait, decolonization therapy is only transiently effective [20]. Importantly, there remains a critical need for measurable and durable biomarkers to predict AECOPD risk. In this study, we characterized the nasal bacterial microbiome at the species level in individuals with COPD, stratified by AECOPD history, and compared findings to healthy controls without lung disease. Longitudinal follow-up further identified nasal microbiome features that were predictive of future AECOPD events.

## Methods

### Participant recruitment

This was a longitudinal study of COPD individuals with or without AECOPD in the 12 months prior to enrollment, and healthy non-COPD comparator participants. Individuals with COPD were enrolled from the University of North Carolina at Chapel Hill Pulmonary Specialty Clinic from November 16, 2021, through October 18, 2023. The study was reviewed and approved by the University of North Carolina at Chapel Hill Institutional Review Board (IRB # 20–3512). Key inclusion criteria for the COPD cohort included age >40 years old, spirometry-confirmed obstruction, history of tobacco use, and no exacerbation in the 4 weeks prior to enrollment. Key exclusion criteria included current use of azithromycin, chronic immunosuppression, intranasal corticosteroids, or supplemental oxygen beyond nocturnal use. Healthy comparator participants were recruited from a healthy volunteer registry and community recruitment. Key inclusion criteria for healthy participants included age >40 years old, normal lung function defined by spirometry, and non-smoker status. Healthy participants had identical exclusion criteria as COPD participants. Complete inclusion and exclusion criteria are available in an online data supplement.

### Study procedures

Nasal epithelial lining fluid (NELF) samples were collected from all participants as previously described [21, 22]. Briefly, participants’ nostrils were moisturized with 100 µL of saline, a Leukosorb strip was introduced into each nare, and the nares were clamped for 2 min. After collection, Leukosorb strips were stored at −80 °C until batch processing for 16S rRNA gene sequencing. After initial sampling, participants were contacted every 3 months for 1 year, and medical charts were reviewed to determine subsequent AECOPD occurrence. Criteria for determining prospective AECOPD are detailed in Supplemental Material.

### Nasal full-length bacterial 16S rRNA gene sequencing

DNA was extracted from a single Leukosorb strip per subject as previously described [22, 23], and full-length bacterial 16S genes were PCR amplified and sequenced using Oxford Nanopore technology. DNA extracted from five Leukosorb strips, spiked with ZymoBIOMICS microbial community standards (Zymo Research), were used as positive controls to verify appropriate taxa assignment. Eight unused Leukosorb strips were processed as negative controls. Reads from the negative controls were used for decontamination. All reads that passed QC were deposited in the publicly available Sequence Read Archive under the Bioproject ID PRJNA1172732. 16S library prep, quality checks, data processing, decontamination, and analysis are described in detail in the online supplement.

### Measurement of Dolosigranulum pigrum abundance

Quantitative polymerase chain reaction (qPCR) was used to target the *murJ* gene of *Dolosigranulum pigrum* (*D. pigrum*) using previously validated primers [24]. Detailed methods are available in the online supplement.

### Hierarchical clustering analysis of the nasal microbiome

Initially low abundance taxa (taxa less than 1% relative abundance) were filtered out. The dataset was centered-log ratio (CLR) transformed. Unsupervised k-means clustering was performed, and the highest average silhouette width was used to determine the optimal cluster number that best represents the microbiome variability in the study population. Individual samples were assigned to one of the two k-means clusters for subsequent analyses. Shannon index and permutational multivariate analysis of variance (PERMANOVA) were used to determine alpha and beta diversity differences between clusters, respectively. Analysis of composition of microbiome with bias correction (ANCOM-BC2) was used to identify taxa which were significantly different between microbiome clusters.

### Microbial network analysis

CLR-transformed species-level abundances were used to compute Pearson-based microbial networks from shared differentially abundant taxa, as previously described [22]. Additional details are available in the online supplement.

### Statistical analysis

For all analyses, the COPD participants were categorized as “Ever AECOPD” if they reported one or more AECOPD in the prior year, and “Never AECOPD” if they reported no AECOPD in the prior year. Clinical and demographic characteristics were compared with summary statistics. Prospective AECOPD and *Dolosigranulum pigrum* threshold analysis was conducted by Mann–Whitney comparison.

## Results

From November 16, 2021, through October 18, 2023, a total of 41 COPD participants and 15 healthy comparators were enrolled. Among the COPD participants, 16 (39%) experienced one or more AECOPD in the year prior to enrollment and were assigned to the Ever AECOPD group. COPD participants without a history of AECOPD in the prior year (*n* = 25) were assigned to the Never AECOPD group. The baseline demographic and clinical characteristics of participants are shown in Table 1. The Never AECOPD group had a mean (SD) age of 68.2 (9.1) years; 52% were female, and 88% were White. A total of 29% were current smokers. The mean (SD) percent predicted FEV1 among COPD without prior AECOPD was 61% predicted. The Never AECOPD group was on diverse inhaler regimens of short-acting bronchodilators only, single or dual bronchodilators, and inhaled steroid (ICS)-bronchodilators. Compared to the Never AECOPD group, the Ever AECOPD group had similar age, sex distribution, and smoking status. Those with prior AECOPD had lower lung function and were more likely to be on an inhaler regimen with LAMA/LABA/ICS (Table 1). Among the Ever AECOPD group, the median number (range) of AECOPD events in the prior year was 2 (1–3). For healthy participants, the mean (SD) age was 57.2 (10.6) years; 67% were female, and 80% were White. As expected by study design, healthy participants had normal % predicted FEV1 (mean 101.8% predicted).

**Table 1:** Baseline demographic and clinical characteristics

	Healthy	Never AECOPD	Ever AECOPD	p**
*N*	15	25	16	-
Age, years	57.2 (10.6)	68.2 (9.1)	68.8 (8.4)	<0.01
Female sex	10 (67)	13 (52)	6 (50)	0.62
Race				
White	12 (80)	22 (88)	14 (88)	0.75
Black	3 (20)	2 (8)	2 (12)	
Asian	0 (0)	1 (4)	0 (0)	
Smoking status*				
Current	0 (0)	7 (29)	5 (31)	<0.01
Former	3 (20)	17 (71)	11 (69)	
Never	12 (80)	0 (0)	0 (0)	
Smoking, pack-years	17.0 (2.6)	35.2 (19.2)	42.4 (17.2)	0.02
Inhaled therapies				
None	14 (93)	1 (4)	0 (0)	<0.01
SABD only	0 (0)	4 (16)	1 (6)	
LAMA	0 (0)	1 (4)	0 (0)	
LABA/ICS	1 (7)	2 (8)	0 (0)	
LABA/LAMA	0 (0)	7 (28)	4 (25)	
LAMA/LABA/ICS	0 (0)	10 (40)	11 (69)	
FEV1, post-BD				
Absolute, Liters	2.79 (0.4)	1.61 (0.7)	1.55 (0.7)	<0.01
% predicted	101.8 (10.8)	61.0 (18.0)	54.3 (17.5)	<0.01
FEV1/FVC, post-BD	0.78 (0.09)	0.51 (0.13)	0.49 (0.12)	<0.01
GOLD Stages				
Stage 1	N/A	3 (12)	2 (13)	0.03
Stage 2		16 (64)	5 (31)	
Stage 3		4 (16)	9 (56)	
Stage 4		2 (8)	0 (0)	
AECOPD in last year, median (range)	N/A	0 (0–0)	2 (1–3)	<0.01

All values mean (SD) or *n* (%) unless otherwise indicated

*Definition of abbreviations: AECOPD *acute exacerbation of COPD, *SABD *short-acting bronchodilator, *LAMA *long-acting muscarinic antagonist, *LABA *long-acting beta-agonist, *ICS* inhaled corticosteroid, *FEV1 *forced expiratory volume in 1 s, *BD *bronchodilator. Never AECOPD = COPD without AECOPD in the prior year; Ever AECOPD = COPD with AECOPD in the prior year

GOLD Stages (based on FEV₁ % predicted): 1 = ≥ 80; 2 = 50 to <80; 3 = 30 to <50; 4 = less than 30

* 1 Missing value in the COPD without prior AECOPD group

**p value calculated by Fisher, Mann–Whitney or Kruskal–Wallis test as appropriate

### Differential bacterial microbiome by clinical/demographic characteristics

Full-length bacterial 16S profiling from nasal Leukosorb strips was performed and compared to demographic and clinical characteristics within the study population. We did not find statistically significant differences in Shannon diversity index or bacterial composition by sex (Figures S1A-B), smoking status (Figures S1C-D), % predicted FEV1 > or ≤ 50 (Figures S1E-F), COPD diagnosis (Figures S1G-H), and age (Figure S1I-J). 

### Nasal bacterial microbiome composition by AECOPD history

**Fig. 1:**
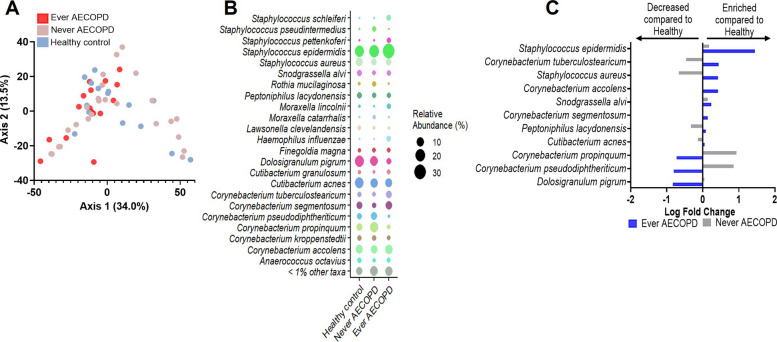
Nasal microbiome by COPD and prior AECOPD. **A** Principal coordinate analysis using Euclidean distance showing the distribution of the bacterial microbiome composition in all subjects categorized by prior AECOPD history. Pairwise PERMANOVA; Ever AECOPD vs Healthy controls (*p* = 0.048), Never AECOPD vs Healthy controls (*p* = 1.000), Ever AECOPD vs Never AECOPD (*p* = 0.180). **B** Relative abundance analysis at the species level comparing taxa composition by prior AECOPD history. The size of the dot is based on taxon relative abundance. **C** Differential abundance analysis (ANCOM-BC2) adjusted by sex and age using taxa above 1% in relative abundance to assess the enrichment or decrease of taxa relative abundance compared to the healthy control group used as reference

Although no statistical difference in Shannon alpha diversity index was observed between AECOPD groups (Ever AECOPD versus Never AECOPD) and healthy controls (Figure S2A), we found significantly different taxa distribution between Ever AECOPD and healthy controls (PERMANOVA, *p* = 0.048) (Fig. 1A and Figure S2B). Differential abundance analyses showed a trend for several highly abundant taxa, including an increase in *Staphylococcus epidermidis* and a decrease in *Dolosigranulum pigrum* in the Ever AECOPD group compared to healthy controls (Fig. 1B, C).

### Hierarchical clustering analysis of the nasal bacterial microbiome

**Fig. 2:**
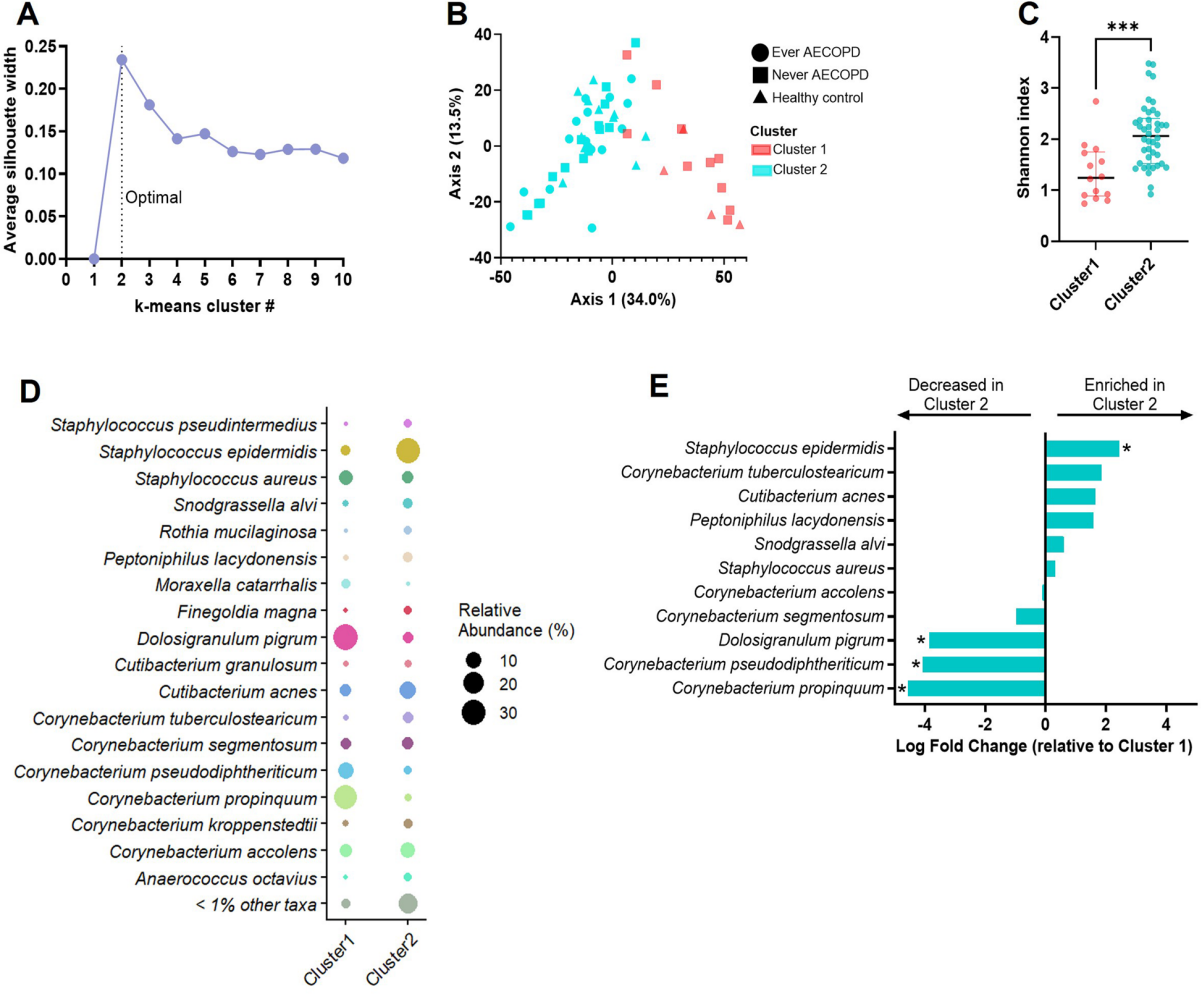
Unsupervised hierarchical clustering nasal microbiome analysis. **A** Silhouette plot showing the average silhouette width based on the number of clusters used to explain the microbiome variability across the study population. Dotted line showing the highest average silhouette statistic, which determined the optimal number of clusters. **B** Principal coordinate plot from Fig. 1A, re-labeled to show the distribution of the nasal microbiomes based on prior AECOPD and k-means clusters. PERMANOVA comparing Cluster 1 and Cluster 2 (*p* < 0.001). **C** Alpha diversity Shannon index comparing bacterial diversity by clusters (*t*-test, *p* < 0.001). **D** Relative abundance comparing taxa distribution by clusters at the species level. The size of the dot is based on taxon relative abundance. **E** Differential abundance analysis of taxa (at the species level) enriched or decreased in Cluster 2 compared to Cluster 1 (reference line). Only taxa above 1% relative abundance were included. ANCOM-BC2 statistic adjusted by sex and age where *q* < 0.05 is marked with an asterisk

We observed that the community structure of the Never AECOPD microbiomes showed a wide distribution that overlapped both the Ever AECOPD and healthy groups (Fig. 1A and Figure S2C), suggesting that the Never AECOPD group was highly heterogeneous. To further explore this, we performed an unsupervised k-means clustering of the microbiome data, which indicated that two clusters best represent the variability of the microbiome across the entire study population (Fig. 2A). Further analysis revealed that Cluster 1 included individuals from the Never AECOPD and healthy control groups, but not Ever AECOPD. Cluster 2 included individuals from all groups (Fig. 2B). Individuals within Cluster 1 had significantly less bacterial diversity compared to Cluster 2 (Shannon Diversity, *p* < 0.001) (Fig. 2C) and the bacterial community composition was significantly different between clusters (PERMANOVA, *p* < 0.001) (Fig. 2B, D). Differential abundance analysis indicated that Cluster 1 is defined by significant enrichment of protective taxa (healthy signature) including *D. pigrum* [14], *Corynebacterium pseudodiphtheriticum* [25]*,* and *Corynebacterium propinquum* [26] (Fig. 2E). In contrast, Cluster 2 is defined by enrichment of pathobionts (pathogenic signature), including *Staphylococcus epidermidis* [27]*, Cutibacterium acnes* [28], *and Staphylococcus aureus* [29] (Fig. 2E), reminiscent of the trend found in our comparison of the Ever AECOPD and healthy groups (Fig. 1C). Finally, re-examination of the heterogeneous Never AECOPD group revealed a significant separation based on our unsupervised clustering assignments (PERMANOVA, *p* < 0.001) (Figure S3A). Compositional similarity analysis using Bray–Curtis dissimilarity index showed that Never AECOPD Cluster 1 subjects were significantly less dissimilar to healthy controls than to Ever AECOPD subjects. In contrast, Never AECOPD Cluster 2 subjects were significantly less dissimilar to Ever AECOPD subjects than to healthy controls (Figures S3B-C). These results suggest that Never AECOPD Cluster 1 subjects are more similar to healthy subjects and Never AECOPD Cluster 2 subjects are more similar to Ever AECOPD subjects.

### Association of nasal bacterial microbiome with future AECOPD

**Fig. 3:**
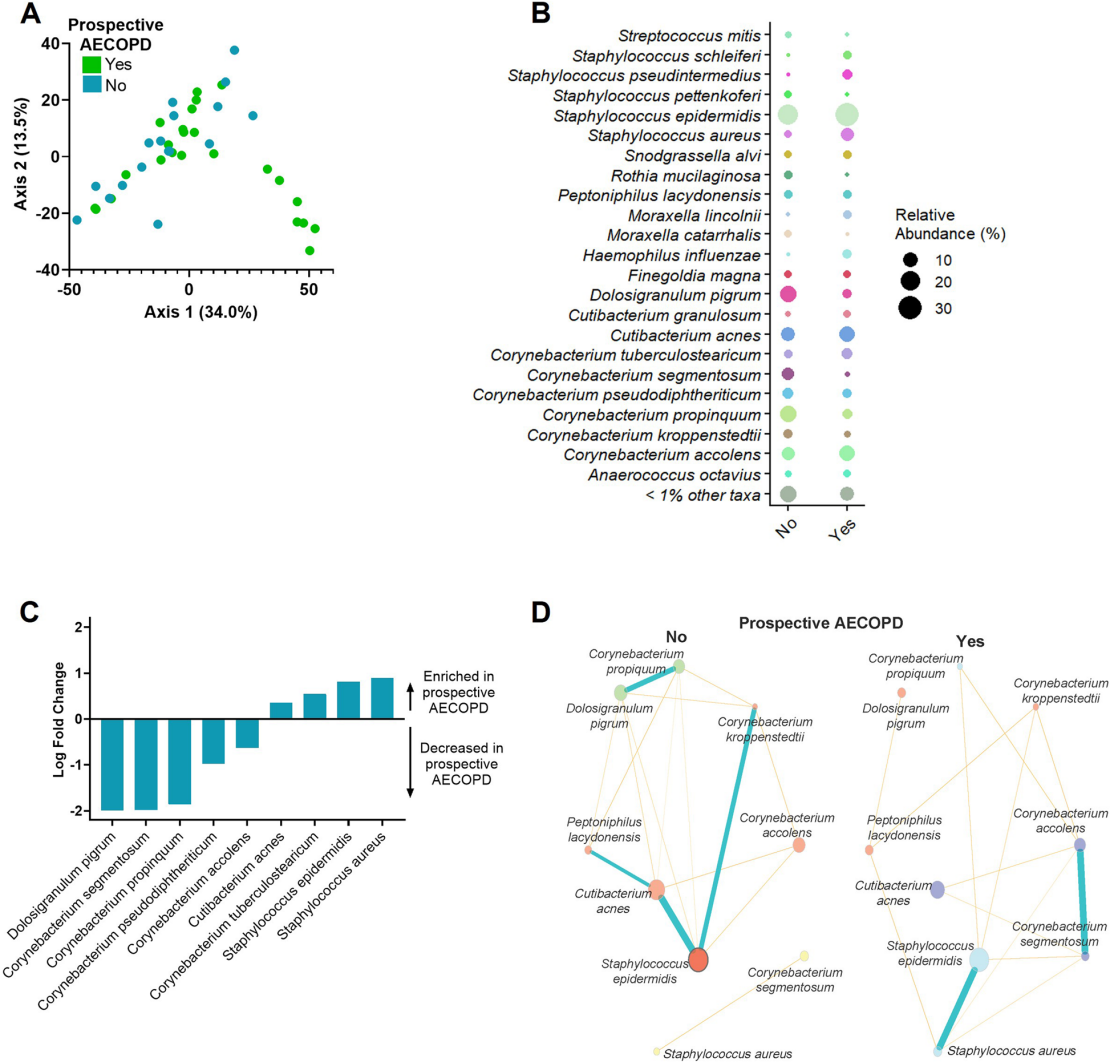
Nasal microbiome by prospective AECOPD. **A** Principal coordinate analysis using PERMANOVA statistics (*p* = 0.04) comparing COPD individuals (*n* = 41) by prospective AECOPD (AECOPD that occurred up to 1 year after sample collection). **B** Relative abundance plot comparing species distribution by prospective AECOPD. **C** Differential abundance analysis (ANCOM-BC2 adjusted by sex) comparing species with greater than 1% relative abundance in COPD individuals based on prospective AECOPD. **D** Microbial network analysis using NetCoMi and Pearson correlation statistics to determine correlation between the most abundant species stratified by prospective AECOPD groups

In a 1-year follow-up, a total of 17 COPD participants (41.5% of COPD cohort) experienced a prospective AECOPD event. The clinical characteristics of the COPD participants stratified by prospective AECOPD are summarized in Table 2. Participants experiencing prospective AECOPD were more likely to be female and have lower lung function at enrollment. Bacterial composition analysis of COPD-diagnosed subjects revealed significant differences in taxa distribution between individuals who experienced at least one prospective AECOPD event after baseline sample collection as compared to participants who did not experience a prospective AECOPD (PERMANOVA, *p* = 0.04) (Fig. 3A). We observed that individuals who experienced prospective AECOPD had lower relative abundance of *D. pigrum, C. segmentosum, and C. propinquum* and higher relative abundance of *Staphylococcus aureus* than individuals who did not experience prospective AECOPD(Fig. 3B, C). A microbial network analysis by prospective AECOPD showed that within COPD subjects without prospective AECOPD, *D. pigrum* positively correlates and clusters together with *Corynebacterium propinquum* while *Staphylococcus epidermidis* positively correlates with *Cutibacterium acnes* and *Corynebacterium kroppenstedtii*. In contrast, in subjects with prospective AECOPD, *Staphylococcus epidermidis* clustered and positively correlated with *Staphylococcus aureus* (Fig. 3D). A logistic regression analysis showed that % predicted FEV1 ≤ 50, prior AECOPD, and total abundance (measured by qPCR) of *D. pigrum* are independently associated with prospective AECOPD (Table 3). Finally, we observed that within the Never AECOPD group, 5/15 (33%) individuals in Cluster 2 and 2/10 (20%) individuals in Cluster 1 experienced prospective AECOPD (Figure S3A).

**Table 2:** Demographic and clinical characteristics of COPD participants stratified by prospective AECOPD event

	COPD without prospective AECOPD event	COPD with prospective AECOPD event
*N*	24	17
Age, years	67.4 (9.5)	69.8 (7.1)
Female sex	11 (46)	7 (58)
Race		
White	20 (83)	16 (94)
Black	3 (13)	1 (6)
Asian	1 (4)	0 (0)
Smoking status*		
Current	8 (33)	4 (25)
Former	16 (67)	12 (75)
Never	0 (0)	0 (0)
Smoking, pack-years	35.4 (15.4)	41.8 (22.2)
Inhaled therapies		
None	1 (4)	0 (0)
SABD only	5 (21)	0 (0)
LAMA	0 (0)	1 (6)
LABA/ICS	1 (4)	1 (6)
LABA/LAMA	6 (25)	5 (29)
LAMA/LABA/ICS	11 (46)	10 (59)
FEV1, post-BD		
Absolute, Liters	1.8 (0.7)	1.3 (0.6)
% predicted	63.7 (16.5)	51.0 (17.6)
FEV1/FVC, post-BD	0.53 (0.12)	0.46 (0.13)
AECOPD in year prior to enrollment, median (range)	0 (0–2)	1 (0–3)

All values mean(SD) or *n*(%) unless otherwise indicated

*Definition of abbreviations: AECOPD *acute exacerbation of COPD, *SABD *short-acting bronchodilator, *LAMA *long-acting muscarinic antagonist, *LABA *long-acting beta-agonist, *ICS *inhaled corticosteroid, *FEV1 *forced expiratory volume in 1 s; *BD *bronchodilator

*1 Missing value in the COPD with prospective AECOPD event group

**Table 3:** Multiple logistic regression of predictors associated with prospective AECOPD

Factor		Bivariate			Multivariate**		
		OR	95% CI	*p*	OR	95% CI	*p*
Age (years)		1.03	0.96–1.11	0.37	---	---	---
Sex							
	Female	---	---	ref	---	---	---
	Male	0.59	0.17–2.08	0.41	---	---	---
Smoking status*							
	Former	---	---	ref	---	---	---
	Current	0.67	0.16–2.74	0.57	---	---	---
% predicted FEV1		0.95	0.92–1.00	0.03	---	---	---
% predicted FEV1 (categories)							
	>50%	---	---	ref	---	---	ref
	≤50%	5.43	1.37–21.6	0.02	3.77	0.83–17.17	0.09
Past AECOPD							
	No	---	---	---	---	---	ref
	Yes	4.29	1.12–16.31	0.03	2.35	0.53–10.54	0.26
qPCR (*D. pigrum*)							
	Negative	---	---	ref	---	---	---
	Positive	0.26	0.07–1.03	0.06	---	---	---
*D. pigrum* log10 (copies/sample)		0.49	0.26–0.92	0.03	0.53	0.26–1.09	0.08
Bacterial cluster							
	Cluster 1	---	---	---	---	---	---
	Cluster 2	3.75	0.68–20.57	0.13	---	---	---

Outcome = Prospective AECOPD (*n* = 41), exclude Healthy controls

*1 Missing value

**Model adjusted by % predicted FEV1 (categories), Past AECOPD, and *D. pigrum* log10 (copies/sample)

### Association between baseline D. pigrum with future AECOPD

**Fig. 4:**
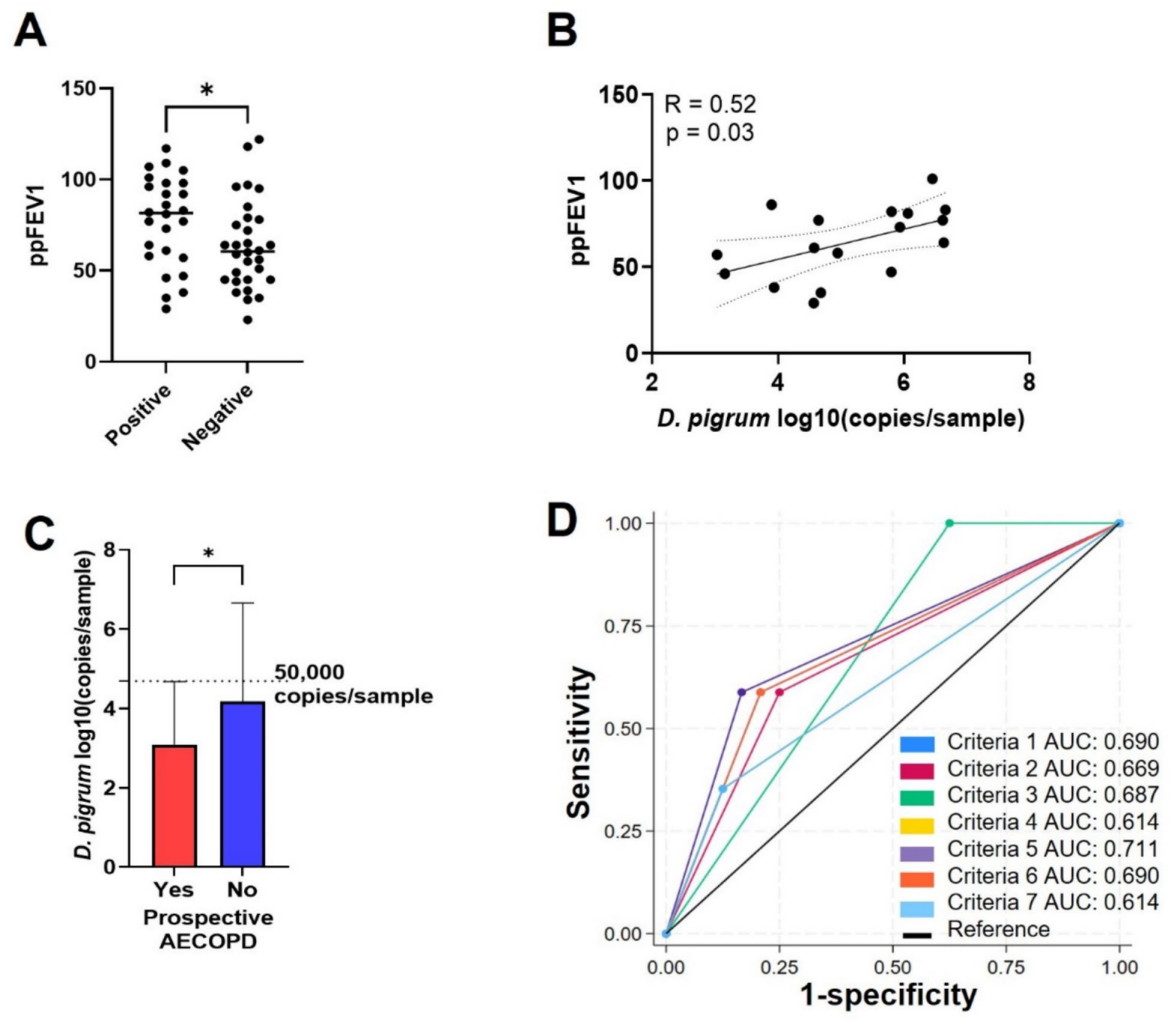
*D. pigrum* qPCR as a predictor of future AECOPD. **A** Comparison of % predicted FEV1 values based on *D. pigrum*-specific qPCR results (positive vs negative amplification, *t*-test *p* = 0.04). **B** Pearson correlation between % predicted FEV1 and *D. pigrum* log10(copies/sample) in individuals with positive *D. pigrum*-specific qPCR. **C** qPCR measured *D. pigrum* log10 (copies/sample) by prospective AECOPD. Dotted line shows that the maximum value for individuals with prospective AECOPD was not over 50,000 genome copies/sample (Mann–Whitney test, *p* = 0.02). **D** Receiver operating characteristic (ROC) analysis comparing different criteria (based on area under the curve) as predictors of prospective AECOPD. Criteria; (1) % predicted FEV1 < 50, (2) prior history of AECOPD, (3) *D. pigrum* < 50,000 copies/sample, (4) % predicted FEV1 < 50 and prior history of AECOPD, (5) % predicted FEV1 < 50 and *D. pigrum* < 50,000 copies/sample, (6) prior history of AECOPD and *D. pigrum* < 50,000 copies/sample, and (7) % predicted FEV1 < 50 and prior history of AECOPD and *D. pigrum* < 50,000 copies/sample

Previous reports indicate that *D. pigrum* is a protective member of the nasal bacterial microbiome [14]. Based on our observations that the relative abundance of *D. pigrum* is lower in participants with prior AECOPD (Fig. 1B, C), differentiates pathogenic and healthy nasal bacterial signatures (Fig. 2D, E), and is specifically associated with prospective AECOPD (Table 3), we evaluated the absolute abundance of *D. pigrum* as a predictor of future exacerbations. *D. pigrum*-specific qPCR indicated that individuals with detectable *D. pigrum* had higher % predicted FEV1 compared to qPCR negative participants (*p* = 0.04) (Fig. 4A). Within the *D. pigrum* qPCR positive individuals, we found a positive correlation between the abundance of *D. pigrum* and % predicted FEV1 (Fig. 4B). Additionally, we observed that all individuals who had a prospective AECOPD had significantly less *D. pigrum* total abundance than individuals with no prospective AECOPD (*p* = 0.02) (Fig. 4C). We also observed that all subjects with prospective AECOPD had less than 50,000 copies of *D. pigrum* per sample (Fig. 4C), which led us to assign two comparable groups (≤50,000 and >50,000 copies/sample).

To evaluate the ability of known clinical markers (% predicted FEV1 < 50 and previous exacerbation history) and our novel biomarker (*D. pigrum* ≤ 50,000 copies/sample) measured at baseline to correctly classify the participants who had/did not have a prospective AECOPD event, we performed a receiver operating characteristic (ROC) analysis. Each criterion, including (1) % predicted FEV1 < 50, (2) history of previous AECOPD, and (3) *D. pigrum* < 50,000 copies/sample, individually had value as a predictor of prospective AECOPD with area under the curve (AUC) of 0.690 (95%CI: 0.54–0.84), 0.669 (95%CI: 0.52–0.82), and 0.687 (95%CI: 0.59–0.79), respectively. Furthermore, using both % predicted FEV1 < 50 and *D. pigrum* < 50,000 copies/sample criteria, we observed the highest AUC values (AUC: 0.711, 95%CI: 0.57–0.85), which were more predictive than % predicted FEV1 < 50 with history of previous AECOPD (AUC: 0.614, 95%CI: 0.48–0.75), or the three predictors combined (AUC: 0.690, 95%CI: 0.54–0.84) (Fig. 4D).

## Discussion

In this prospective observational analysis of the nasal bacterial microbiome of individuals with COPD and varying AECOPD history, we observed several key findings. First, we identified nasal microbiome signatures that can be classified as either pathogenic or healthy. The pathogenic signature uniquely identifies individuals with prior AECOPD history. Second, COPD individuals with prior AECOPD history have a distinct nasal microbiome compared to COPD individuals with no AECOPD history or healthy comparators. Finally, reduced abundance of nasal *D. pigrum* was associated with % predicted FEV1 and prospective AECOPD. Together these data identify the nasal microbiome as a putative biomarker of AECOPD risk and a potential target for disease modifying therapies.

We found significantly distinct nasal microbiome signatures that divided our population into two microbial clusters, with Cluster 1 representing healthy controls and COPD subjects from the Never AECOPD group (healthy cluster) and Cluster 2, enriched for COPD subjects with a history of AECOPD (pathogenic cluster). In addition, we observed that the healthy cluster (Cluster 1) had statistically significant lower alpha diversity and was significantly enriched for previously reported protective taxa, *Dolosigranulum pigrum* [14]*, Corynebacterium propinquum, and Corynebacterium pseudodiphtheriticum* [26] compared to the pathogenic cluster. Traditionally, lower alpha diversity is associated with poor outcomes and/or the presence of pathogens; however, in the nasal microbiome, we observed the opposite. We hypothesize that the dominance of key protective commensals (i.e., gatekeepers) in the healthy nasal microbiome may limit colonization by other taxa, thus restricting microbial diversity. A reduction in beneficial taxa may allow opportunistic taxa and potentially pathogenic species to colonize, driving higher diversity.

Importantly, we observed that *D. pigrum* abundance is higher in healthy controls than in subjects with COPD. Within the COPD cohort, those with a prior history of AECOPD had the lowest abundance of *D. pigrum* (Fig. 1). Our data show that participants who are qPCR positive for *D. pigrum* have significantly higher % predicted FEV1 compared to participants with undetectable *D. pigrum* and that *D. pigrum* abundance potentially has individual and additive value (when used together with the % predicted FEV1 ≤ 50 criteria) to predict future exacerbation in COPD patients (Fig. 4).

Several studies have shown that the abundance of *D. pigrum* inversely correlates with respiratory symptoms and several upper and lower respiratory diseases [30–32]. The strong association between *D. pigrum* abundance and prospective AECOPD indicates that the nasal microbiome may be a treatable trait and potential target for therapeutic intervention. While the gut microbiome and the gut-lung axis have been explored as a target for supplementation with probiotics and/or prebiotics to modify COPD, exploration of nasal probiotic administration is much more limited [33–35]. *D. pigrum* has been proposed as a promising probiotic for respiratory health in other respiratory disease settings [14]. Our results suggest that this benefit may also extend to COPD.

The mechanism by which *D. pigrum* provides protection remains to be determined, but it has been well established that *D. pigrum* can directly inhibit the growth of pathogens including Staphylococcal species, *Moraxella catarrhalis*,* and Streptococcus pneumoniae* [14, 36, 37]. While *D. pigrum* has an unusually small genome with limited metabolic capabilities, it encodes an abundance of biosynthetic gene clusters predicted to encode antimicrobial compounds and bacteriocins that may account for its protective properties [36, 37]. It is also possible that the presence of *D. pigrum* may suppress the levels of inflammatory cytokines, providing some mitigation against a future exacerbation. Alternatively, *D. pigrum* may not provide direct protection, but rather thrive in a healthy airway. In this case, loss or reduction of *D. pigrum* may be due to increased inflammation or enrichment of pathogens that increase the risk of AECOPD. This alternative interpretation does not reduce the value of *D. pigrum* as a predictor of AECOPD.

We observed additional differences in the nasal microbiome based on prospective COPD exacerbation. Specifically, *S. aureus* and *S. epidermidis* were enriched in Ever-AECOPD participants compared to healthy controls, and similarly in COPD Cluster 2 (pathogenic) compared to Cluster 1 (healthy). However, *S. aureus* enrichment was not statistically significant in any analysis. *S. epidermidis,* a widely studied pathobiont, which can be commensal or pathogenic [27], was significantly enriched in groups at higher risk of exacerbation, but its utility as a biomarker is inherently limited. *S. epidermidis* abundance can vary substantially between individuals, and levels are already high in healthy subjects. Furthermore, *S. epidermidis* is one of the most abundant members of the skin microbiota and a frequent contaminant of nasal and airway sampling, making it difficult to distinguish true biological signal from contamination or transient colonization.

In COPD individuals without prospective AECOPD, *S. epidermidis* positively correlated with *Corynebacterium* and *Cutibacterium* species. However, in individuals with prospective AECOPD, *S. epidermidis* positively correlated with *S. aureus* (Fig. 3). This may partially reflect a shift in *S. epidermidis* behavior from beneficial commensal to pathogen that can exist as a symbiont with *S. aureus*. Previous studies have shown mutual commensal cooperation of *Dolosigranulum* and *Corynebacterium* species that negatively correlate with *Staphylococcus* and *Streptococcus* abundance [36]. Our findings demonstrate that a substantial decrease in *D. pigrum* abundance is associated with future exacerbations in COPD. The association between *D. pigrum* and prospective AECOPD can be partially explained by the relationship between % predicted FEV1 and *D. pigrum;* in fact, high abundance of *D. pigrum* may be an indicator of healthy pulmonary function (FEV1); however, the additive effect of *D. pigrum* abundance and % predicted FEV1 to predict future exacerbations suggests that FEV1 does not fully explain the role of *D. pigrum* as a predictor of prospective AECOPD.

### Limitations

This study has limitations. The population was recruited from a single site with over-representation of Caucasian race. It is possible that geographic or racial diversity may lead to different results. The small sample size predisposes to underpowering to detect other potential associations. Initial ascertainment of AECOPD events relied on self-report, which can be subject to recall bias. Despite this, the findings are novel and establish the rationale for further study of the nasal mucosal environment in COPD. Our study shows no statistical differences in nasal microbiome based on demographic/behavioral characteristics such as sex and smoking status. However, sex-based differences in nasal bacterial microbiome have been identified before by us and others [22]. This can be explained by the low sample size of this study that may impact statistical estimates. We do believe that sex could be a confounding variable in nasal microbiome studies. To address this, previous studies have done sex-matched comparisons [38]; however, given our sample size limitation, we performed variable adjustment by sex in all our differential abundance analyses. We did collect data related to current use of azithromycin as an exclusion criterion but did not collect previous antibiotic exposure, due to incomplete medical history on all participants. Previous antibiotic exposure could affect microbiome metrics, impacting our results in an unpredictable manner; however, all participants had to be free of respiratory infection (cough, sore throat, sinusitis, fever) for 4 weeks prior to enrollment, which decreased the chance they had recent antibiotic exposure for respiratory reasons. The differences on prior AECOPD by inhaled therapies may also impact our results; however, the limited size of our cohort combined with the diversity of therapies used precludes appropriate adjustment. A larger multi-site study is needed to clarify the effects of each inhaled therapy on COPD exacerbations and the nasal microbiome.

## Conclusion

In conclusion, we have shown that the nasal bacterial microbiome in patients with COPD differs significantly between those with and without a history of AECOPD. These findings not only suggest that AECOPD patients have altered bacterial communities in the nasal mucosa but also point to a potential treatable trait to reduce the risk for future AECOPD events. Specifically, the bacterium *D. pigrum* is found in higher abundance in COPD individuals without prior AECOPD events and was predictive of future AECOPD events. This observational study suggests that the absolute abundance of *D. pigrum* could serve as a biomarker to determine the risk of future exacerbation in COPD-diagnosed individuals. Our findings need to be confirmed and validated in a larger independent cohort to fully establish whether *D. pigrum* can be used as a biomarker alone (in areas where spirometry is not possible or widely available) or broadly in combination with spirometry to determine AECOPD risk.

## Supplementary Information

Supplementary Material 1.

## Data Availability

The datasets generated and/or analyzed during the current study are available in the publicly available Sequence Read Archive under the Bioproject ID PRJNA1172732.
