# Supplementary material for: Dysbiosis of the nasal microbiome is associated with prospective acute exacerbation of COPD
Source: Microbiome. 2026 Mar 16;14:120. doi: 10.1186/s40168-026-02357-1 (PMC13104320; doi:10.1186/s40168-026-02357-1)
Supplement: Supplementary file 2 — Supplementary Material 1. [file 40168_2026_2357_MOESM1_ESM.docx]

Supplementary Materials

**Methods**

**Participant Screening, Enrollment and Study Procedures**

All participants were approached in clinic or contacted via phone for screening using an IRB-approved screening script. If eligible and interested, participants attended an in-person screening visit for complete eligibility assessment.

Baseline assessment including a review of medical history and medications and self-reported exacerbation history in prior 12 months (for COPD participants). Screening spirometry was performed using handheld spirometer (if clinical spirometry data from the prior 30 days was not available) following ATS standards. All research coordinators were certified on spirometry procedures and spirograms were reviewed by study pulmonologist for quality.

**Participant Inclusion and Exclusion Criteria**

*COPD cohort*

Inclusion criteria:

- Age>40 years old
- Physician diagnosis of COPD confirmed by post-bronchodilator testing (defined as FEV1/FVC< lower limit of normal and FEV1/FVC<0.70) from spirometry obtained within 30 days of screening
- History of tobacco use (at least 200 cigarettes in participant’s lifetime)
- At the time of screening, free of acute exacerbation of COPD for prior four weeks

Exclusion criteria:

- Current use of azithromycin, chronic immunosuppression or intranasal corticosteroids
- Supplemental oxygen use beyond nocturnal oxygen therapy
- History of Severe Acute Respiratory Syndrome Coronavirus 2 (SARS-CoV-2) infection requiring hospitalization
- History of epistaxis, easy bleeding, current blood thinner use, or prior nasal surgery or anatomical abnormalities increasing risk of nasal sampling

*Healthy cohort*

Inclusion criteria:

- Age>40 years old
- Normal lung function defined as pre- and post-bronchodilator FEV1/FVC>= lower limit of normal and FEV1>80% predicted within 30 days of screening
- Non-smoker (defined as having no cigarette use in the last 10 years).

Exclusion criteria:

- Current use of azithromycin, chronic immunosuppression or intranasal corticosteroids
- Supplemental oxygen use beyond nocturnal oxygen therapy
- History of Severe Acute Respiratory Syndrome Coronavirus 2 (SARS-CoV-2) infection requiring hospitalization
- History of epistaxis, easy bleeding, current blood thinner use, or prior nasal surgery or anatomical abnormalities increasing risk of nasal sampling

**Nasal Full-length Bacterial 16S rRNA Gene Sequencing**

DNA was extracted from whole Leukosorb strips using the DNeasy PowerSoil Pro kit (Qiagen, Inc.) and eluted in 50µL water. Full-length 16S was PCR amplified from 3µL of the eluted DNA using Q5 Hot Start 2x Master Mix (New England BioLabs), primers 27F (5′-AGRGTTTGATYHTGGCTCAG) and 1492R (5′-CGGYTACCTTGTTACGACTT) [(1)](https://sciwheel.com/work/citation?ids=11514456&pre=&suf=&sa=0&dbf=0) at a final concentration of ~0.04µM each, using the following parameters: 95°C for 30sec, then 25 cycles of 95°C-15sec, 60°C-30sec, 72°C-2min, lastly, a final extension at 72°C for 5 minutes. Amplicons were purified using Ampure XP beads (Beckman Coulter Inc.) at 0.8X. Sequencing libraries were prepared following the manufacturer protocol using the native barcoding kit V.14 (Oxford Nanopore Technologies). Libraries were sequenced on a PromethION flow cell R10.4.1 in a P2solo device (Oxford Nanopore Technologies). Reads with simplex Q score <10, length less than 1kb or greater than 2kb were excluded. Reads that passed QC were used in the data analysis and the files were uploaded to the publicly available Sequence Read Archive under the Bioproject ID PRJNA1172732.

**16S data processing**

Reads were processed using the previously described pipeline “Emu” (2). Briefly, reads were mapped using minimap2 to a 16S reference database composed by NCBI 16S RefSeq (3) and rrnDB version 5.6 (4). Assigned reads were counted and arranged in a taxa-table with taxonomic identifiers. DNA extracted from 5 Leukosorb strips spiked with ZymoBIOMICS microbial community standards (Catalog# D6300) were used as positive controls to verify appropriate taxa assignment. A phyloseq object comprising metadata, taxa-table, and taxonomy identification was built using phyloseq package V.1.48 [(5)](https://sciwheel.com/work/citation?ids=3722481&pre=&suf=&sa=0&dbf=0). To address potential taxa contamination during DNA extraction, 8 unused Leukosorb strips (negative controls) undergo the same DNA extraction and taxa mapping as the study samples, reads from the negative controls were used to decontaminate the study samples using the prevalence method of the package decontam in R [(6)](https://sciwheel.com/work/citation?ids=6157455&pre=&suf=&sa=0&dbf=0). Then, data was rarefied to 20000 reads per sample to eliminate sequencing depth bias. The cleaned and rarefied data was used in the downstream diversity analyses.

**Microbial diversity analyses**

Shannon alpha diversity index was measured from each sample using the phyloseq package in R. Using histograms and Shapiro-wilk test, all diversity index distributions were evaluated for normality. To determine alpha diversity differences between groups, non-parametric tests were used (either Mann-Whitney or Kruskal-Wallis as appropriate) in GraphPad Prism software. Microbial compositional differences were determined by merging taxa abundance based on the corresponding demographic/clinical groups and relative abundance of taxa at the species taxonomic level was calculated. Additionally, principal coordinate analyses based on Euclidean distances were used to estimate spatial taxonomic distribution and statistical differences were measured by the PERMANOVA test. Finally, using the analysis of composition of microbiomes with bias correction (ANCOM-BC2) (7), we measured the differential abundance of each species by subject groups. Variable adjustment to control for confounding effects was also performed using ANCOM-BC2.

**Microbial Network Analysis**

Abundance of taxa at species level was CLR normalized and used to compute a Pearson correlation to determine the correlation between taxa within the same sample group. The microbial network analysis was performed only on the most differentially abundant taxa that were present in both sample groups using the Network Construction and Comparison for Microbiome Data (NetCoMi) package in R (8).

**Measurement of *Dolosigranulum pigrum* abundance**

Quantitative polymerase chain reaction (qPCR) was used to target the *murJ* gene of *Dolosigranulum pigrum* (*D. pigrum*) using previously validated primers (9). A standard curve was obtained using isolated genomic DNA from *D. pigrum* strain NCFB 2975 (ATCC). qPCR was performed using PowerTrack SYBR green master mix (ThermoFisher Scientific Inc.) and cycling/melting conditions 50°C-2min, 95°C-2min, 60°C-30sec, 95°C-1sec, 60°C-20sec, 95°C-1sec.

**Measurement of acute exacerbation of COPD**

This was a self-report variable obtained based on the following questionnaire:

*“Since your last research contact, have you had any worsening breathing requiring antibiotics and/or steroids?”*

*If yes, then asked:*

*What was the approximate start date of the first episode?*

*What was the approximate end date of the first episode?*

*For the first episode, did you take antibiotics?*

*For the first episode, did you take steroids (e.g., prednisone)?*

*For the first episode, did you go to the emergency department?*

*For the first episode, did you get hospitalized?*

We then asked if they had a second exacerbation and collected the same data as above. We also manually reviewed medical records to identify additional exacerbations and abstracted the information above.

**Supplementary References**

1. Abellan-Schneyder I, Matchado MS, Reitmeier S, Sommer A, Sewald Z, Baumbach J, et al. Primer, Pipelines, Parameters: Issues in 16S rRNA Gene Sequencing. mSphere. 2021 Feb 24;6(1).

2. Curry KD, Wang Q, Nute MG, Tyshaieva A, Reeves E, Soriano S, Wu Q, Graeber E, Finzer P, Mendling W, Savidge T, Villapol S, Dilthey A, Treangen TJ. Emu: species-level microbial community profiling of full-length 16S rRNA Oxford Nanopore sequencing data. *Nat Methods* 2022; 19: 845-853.

3. O'Leary NA, Wright MW, Brister JR, Ciufo S, Haddad D, McVeigh R, Rajput B, Robbertse B, Smith-White B, Ako-Adjei D, Astashyn A, Badretdin A, Bao Y, Blinkova O, Brover V, Chetvernin V, Choi J, Cox E, Ermolaeva O, Farrell CM, Goldfarb T, Gupta T, Haft D, Hatcher E, Hlavina W, Joardar VS, Kodali VK, Li W, Maglott D, Masterson P, McGarvey KM, Murphy MR, O'Neill K, Pujar S, Rangwala SH, Rausch D, Riddick LD, Schoch C, Shkeda A, Storz SS, Sun H, Thibaud-Nissen F, Tolstoy I, Tully RE, Vatsan AR, Wallin C, Webb D, Wu W, Landrum MJ, Kimchi A, Tatusova T, DiCuccio M, Kitts P, Murphy TD, Pruitt KD. Reference sequence (RefSeq) database at NCBI: current status, taxonomic expansion, and functional annotation. *Nucleic Acids Res* 2016; 44: D733-745.

4. Stoddard SF, Smith BJ, Hein R, Roller BR, Schmidt TM. rrnDB: improved tools for interpreting rRNA gene abundance in bacteria and archaea and a new foundation for future development. *Nucleic Acids Res* 2015; 43: D593-598.

5. McMurdie PJ, Holmes S. Phyloseq: a bioconductor package for handling and analysis of high-throughput phylogenetic sequence data. Pac Symp Biocomput. 2012;235–46.

6. [Davis NM, Proctor DM, Holmes SP, Relman DA, Callahan BJ. Simple statistical identification and removal of contaminant sequences in marker-gene and metagenomics data. Microbiome. 2018 Dec 17;6(1):226.](https://sciwheel.com/work/bibliography/6157455)

7. Lin H, Peddada SD. Analysis of compositions of microbiomes with bias correction. *Nat Commun* 2020; 11: 3514.

8. Peschel S, Müller CL, von Mutius E, Boulesteix AL, Depner M. NetCoMi: network construction and comparison for microbiome data in R. *Brief Bioinform*. Jul 20 2021;22(4)doi:10.1093/bib/bbaa290

9. Aziz M, Palmer A, Iversen S, Salazar JE, Pham T, Roach K, Becker K, Kaspar U, Price LB, Baig S, Stegger M, Andersen PS, Liu CM. Design and validation of Dolosigranulum pigrum specific PCR primers using the bacterial core genome. *Sci Rep* 2023; 13: 6110.
